# How Family Structure Can Influence Adolescents’ Eating Habits: An Italian Health Behaviour in School-Aged Children (HBSC) 2022 Sample

**DOI:** 10.3390/children11111368

**Published:** 2024-11-12

**Authors:** Bianca Maria Bocci, Dario Lipari, Andrea Pammolli, Rita Simi, Elena Frongillo, Antonella Miserendino, Ilaria Manini, Giacomo Lazzeri

**Affiliations:** 1 Post Graduate School of Public Health, University of Siena, 53100 Siena, Italy; d.lipari@student.unisi.it (D.L.); e.frongillo@student.unisi.it (E.F.); a.miserendino2@student.unisi.it (A.M.); manini3@unisi.it (I.M.); giacomo.lazzeri@unisi.it (G.L.); 2 Department of Molecular and Developmental Medicine, University of Siena, 53100 Siena, Italy; pammolli2@unisi.it (A.P.); rita.simi@unisi.it (R.S.); 3 Research Center on Health Prevention and Promotion (CREPS), University of Siena, 53100 Siena, Italy; 4 Interuniversity Research Centre on Influenza and Other Transmissible Infections (CIRI-IT), 16132 Genoa, Italy

**Keywords:** eating habits, family structure, adolescents, parental influence, survey, Italian Health Behaviour in School-aged Children

## Abstract

Background/Objectives: Adolescence is an important period of psychophysical development. In this phase of life people acquire greater self-awareness and adopt behaviors that will later shape their lifestyle in adulthood. This study aimed to assess whether family structure can influence adolescents’ eating habits. Methods: Data were acquired from a survey titled ‘Italian Health Behaviour in School-aged Children’ (HBSC) given to a representative sample of Tuscan adolescents aged 11, 13, 15 and 17 years of age. Participants (3210) filled out a validated questionnaire answering questions about their eating habits and family structure. After adjusting for covariates, some interesting trends were highlighted in the survey results. Results: Adolescents living in single-mother families reported a lower intake of fruits (OR 0.68, 95% CI = 0.55–0.83) and vegetables (OR 0.66, 95% CI = 0.53–0.81) and a higher intake of sugar-added soft drinks (OR 1.33, 95% CI = 1.08–1.64). Living in a mother and stepfather family was associated with a higher intake of sugar-added soft drinks (OR 1.53, 95% CI = 1.02–2.28), whereas living in a father and stepmother family was associated only with a lower intake of fruits (OR 0.48, 95% CI = 0.26–0.87). Participants living with other family types reported a lower intake of vegetables (OR 0.68, 95% CI = 0.47–0.98). Conclusions: The results of this study show a correlation between family structure and adolescents’ eating habits.

## 1. Introduction

Adolescence represents a key stage in a person’s physical and psychological development, which is why behaviors adopted during this period of life are particularly important, as they have lasting consequences on an individual’s health [1,2,3]. In recent years, new/diverse family forms have increasingly emerged. The traditional family consisting of a father and mother is no longer the only model to be considered, so it is essential to investigate how and to what extent different family structures may affect the nutrition of younger people. The prevalence of adolescent obesity has increased in recent years, and it is of paramount importance to understand its causes to prevent a further increase [4,5,6,7,8,9,10]. The eating behaviors of adolescents are influenced by several factors, among which family is undoubtedly one of the main determinants. Numerous studies have shown the correlation between family socioeconomic status and adolescents’ eating habits. Socio-economic status has been shown to have an impact on the eating habits of adolescents; those living in households with lower SES are more likely to engage in poor eating behaviors, such as frequenting fast-food restaurants more often and eating fewer servings of fruit and vegetables during the week [10,11,12,13]. Parents set examples for their children by making healthy food choices and providing healthy food options [14,15,16,17,18,19,20]. In this context, it is therefore important to investigate the correlation between the family and the eating habits of adolescents. In recent years, family structure has become increasingly complex, undergoing major diversification processes. New family structures have emerged, and it is increasingly possible to come across single-parent families, or parents who have divorced and live with one parent and their partner, or extended families [1,2,3,5]. These changes could have an impact on the psycho-physical development of adolescents, and it is important to study the possible relationship between family structure and adolescents’ lifestyles, among which eating habits play a predominant role [1,2,3,4,5]. However, there is a lack of studies focusing on the correlation between family structure that takes into consideration the new realities that have formed in recent decades, particularly families including a single mother, single father, mother and stepfather, father and stepmother, and other family structures (grandparents or adults other than immediate family, such as foster parents, or care homes) [1,3,5]. The present study aimed to assess the influence that different family structures may have on adolescents’ eating habits, focusing on the ages of 11, 13, 15 and 17, the latter being an age group studied little, and thus a new feature of our study.

## 2. Materials and Methods

The present study reported representative regional data from the Tuscan Region, part of the national collaborative Italian Health Behaviour in School-aged Children survey 2022 [21]. The overall aim of the Health Behaviour in School-aged Children study is to enhance the understanding of young people’s health behaviors in their social settings. In the current study, specific school years were the primary sampling unit and a sample of 11 (877), 13 (879), 15 (751) and 17 (703) year-old schoolchildren (3210) participated. A stratified cluster sampling method was employed to obtain representative samples for each age group, with school classes serving as the primary sampling unit. The sampling strategy was implemented using a complete and alphabetically ordered list of public and private schools in Tuscany. The methodology and data collection procedures were designed to ensure the sample was representative of the adolescent population in Tuscany. Further details on these procedures can be found in separate publications [21,22]. The HBSC survey protocol received approval from the Institutional Ethical Board of the National Institute of Health (General protocol: PRE-876/17).

### 2.1. Measures

To assess the frequency of consumption of fruit, vegetables, sweets and sugar-added soft drinks the following question was posed: How many times a week do you usually eat or drink …? Participants responded by ticking one of seven responses: ‘never’, ‘less than once a week’, ‘once a week’, ‘two to four days a week’, ‘five to six days a week’, ‘once a day, every day’ and ‘every day, more than once’. Response options were recoded into dichotomous outcome variables (“daily” or “less than once a day”).

Socioeconomic status was assessed according to the Family Affluence Scale (FAS), a reliable indicator of family wealth [23]. The scale consists of six questions, including family car ownership, whether adolescents have their own bedroom, the number of holidays taken in the last year, the number of computers owned by the family, dishwasher ownership, and the number of bathrooms in the home. The obtained score (0–13) was recoded in a 3-point ordinal scale according to low (0–6), medium (7–9), and high (≥10) family affluence.

To determine family status, the following was present on the survey: “All families are different (for example, not everyone lives with both their parents, sometimes people live with just one parent, or they have two homes or live with two families) and we would like to know about yours. Please answer this question for the home where you live all or most of the time and tick the people who live there: Mother; Father, Stepmother (or father’s girlfriend/partner), Stepfather (or mother’s boyfriend/partner); I live in a foster or children’s home; with someone or somewhere else (e.g., grandparents)”.

Siblings were assessed by two items referring to the household the participants lived in all or most of the time:

“Siblings: Please say how many brothers and sisters live here (including half, step or foster brothers and sisters). Please write in the number or write 0 (zero) if there are none. Please do not count yourself”. (How many brothers? How many sisters?).

Other family structures include grandparents and adults other than parents, such as foster parents, or care homes.

Body Mass Index (BMI) was calculated using self-reported weight and height (kg/m^2^). BMI scores were recoded into standardized *z*-scores.

### 2.2. Statistics

To evaluate the association between food habits (as a dependent ordinal variable) and family structure (as an independent dummy variable), an ordered logistic regression was used. To adjust the parameter estimate, we put the following variables as covariates into the model: age, gender, BMI, number of siblings and SES, the relative one-way interaction terms to each of these and family structure. Likelihood ratio tests were used to evaluate the significance of the effect (*p* < 0.05). In case of the violation of the proportional odds assumption (necessitating implementation of an ordered logistic regression and a test by the parallels lines test), a multinomial logistic regression was applied in cases where the Akaike information criterion (AIC) or Bayesian information criterion (BIC) for this model was less than the correspondence criteria information assuming proportional odds. All statistical procedures were performed in SPSS version 22.0.

## 3. Results

The sample’s attributes are displayed in Table 1. A little over sixteen percent of participants said they were in step-parent or single-parent households. In general, the highest response frequencies for fruit consumption were “2–4 days a week”, with the exception of eleven-year-olds, where the highest percentages were for the response “5–6 days a week” (Table 2). Approximately 70% of respondents said they ate sweets more than once a week, and 30% said they drank soft drinks with added sugar more than once a week (data not reported in tables).

### 3.1. Associations Between Family Structure and Adolescents’ Food Habits

No adjusted associations were found for fruit and vegetable consumption and single-mother households; soft drinks and mother/stepfather homes; fruit and father/stepmother homes; and vegetables in ‘other family’ scenarios. In the modified models, the correlations held true (Table 3). No statistically significant interaction was found between family structure and the identified factors. Below are further specifics from the adjusted main effects analyses.

### 3.2. Single-Parent Families

According to the adjusted analyses, adolescents from single-mother households reported consuming more sugar-sweetened soft drinks (OR 1.33, 95% CI 1.08–1.64), less fruits and vegetables (OR 0.68, 95% CI 0.55–0.83), and more vegetables overall (OR 0.66, 95% CI 0.53–0.81).

### 3.3. Step-Parent Families

Taking as a reference category “living with both parents”, adolescents living in mother and stepfather families reported consuming more sugar-added soft beverages (OR 1.53, 95% CI 1.02–2.28) according to the adjusted analyses. Only a decreased fruit intake was linked to living in a father and stepmother household (OR 0.48, 95% CI 0.26–0.87).

### 3.4. Other Parent Family

The adjusted analyses showed that adolescents living in other family structures reported consuming less fruit, vegetables and sweets and more soft drinks than their counterparts living with both parents. Living in other family structures was only associated with a lower vegetable intake (OR 0.68, 95% CI 0.47–0.98).

### 3.5. Age and Gender Differences

Table 3 illustrates the correlation between the female gender and higher soft drink intake and lower consumption of vegetables and sweets. The older age groups (15 and 17 year-olds) were linked to higher intakes of vegetables and sweets and lower intakes of fruit.

### 3.6. Socio-Economic Status

Comparing families with higher SES to those with low SES, the adjusted analyses revealed that the former were associated with a lower consumption of sweets (OR 0.78, 95% CI 0.63–0.96) and a higher consumption of fruits (OR 1.56, 95% CI 1.27–1.92) and vegetables (OR 1.59, 95% CI 1.30–1.95).

### 3.7. BMI

The adjusted analysis revealed that an increase in BMI was associated with an increase in the consumption of vegetables (OR 1.13, 95% CI 1.05–1.21) and a decrease in the consumption of sweets (OR 0.77, 95% CI 0.72–0.83) and soft drinks (OR 0.91, 95% CI 0.85–0.97).

### 3.8. General Comments

The assumption of proportional odds was only broken for the consumption of fruits and soft drinks (*p* = 0.03, *p* < 0.001). Ordinal regression was nevertheless utilized, as the AIC and BIC were lower than the correspondence criteria information obtained from the multinomial model.

## 4. Discussion

Our study showed that most adolescents, in line with their Italian and European peers, are far from following WHO recommendations regarding proper eating habits [1,2,3,6,7,8,9,10]. As shown by collected and analyzed data, most adolescents did not consume fruits and vegetables daily, and among them, the consumption of soft drinks with added sugar and sweets was high. Previous studies focused on differences in adolescents’ eating habits related to their gender, age, existing relationship with their parents, and the socioeconomic status of the family in which they live [12,13,14,15,16,17,18,19,20]. Few studies, however, have evaluated the influence that family structure may have on adolescents’ eating habits [1,3,5].

Our analysis showed that more and more adolescents are living in families with structures other than the two-parent family; in fact, our study revealed about 16% live in a single-parent home. In line with trends reported in other studies [1,2,3,4,5], most participants reported not eating fruits and vegetables daily, more than 70% consumed sweets more than once a week, and 30% consumed beverages with added sugar more than once a week.

Our study suggests that adolescents living in single-mother households consumed fewer fruits and vegetables and more soft drinks with added sugars than those living with both parents did. These data are in line with published data from other European studies [1,2,3]. An explanation can be found in the fact that single mothers usually have less time to devote to meal preparation and often belong to classes with lower socioeconomic status. Adolescents living with a mother and stepfather consumed more sugar-added soft drinks than their counterparts living with both parents, but no substantial difference was noted in fruit and vegetable intake. There may be an explanation for these results in the fact that stepfathers may have natural children to care for financially and may have less time on their hands and fewer economic resources than natural fathers [24,25,26,27,28].

Adolescents living with their father and stepmother, on the other hand, had a lower fruit intake than their counterparts living with both parents. Usually, fruit consumption is higher among girls than boys, and adolescent girls emulate their mothers for fruit consumption, which may be an explanation for this phenomenon [23,24,27]. Teenagers living in other households (with grandparents and adults other than parents, such as foster parents, or care homes) consumed fewer vegetables than their peers living with both parents [1,2,3,4,24].

It was also found that females consumed fewer sweets and vegetables and more soft drinks with added sugar than males. As age increased, fruit consumption decreased and vegetable and sweets consumption increased. Higher socioeconomic status was associated with higher consumption of fruits and vegetables and lower consumption of sweets compared to households with a lower socioeconomic status [24,25,26,27,28].

The latter results can be easily explained by the higher cost of fruits and vegetables and their perishability, so it is easy to see how it is cheaper to resort to carbohydrates and sweets for low-income families [25,29,30,31,32,33,34,35,36,37,38,39].

### Strengths and Limitations

There are a few strengths and limitations to consider. This study’s strengths include its use of sizable cross-regional data collection of teenagers from four distinct age groups, its application of standardized procedures, and its ongoing validation of the instruments it uses. However, there has been debate regarding the reliability of adolescents’ self-reported food practices. Furthermore, the food frequency questionnaire only asks about frequency of intake; it does not record the quantity of fruits and vegetables ingested.

Furthermore, there were no classifications of fruits or vegetables given; therefore, interpretations may vary throughout nations. Cross-country comparisons may also be impacted by seasonal bias because access to fruits and vegetables varies by season and data collection times differ between countries. Also, even though the questionnaires were anonymous and students had no reason to feel pressured by peers or society, we cannot rule out the possibility that the increased focus on fruits and vegetables in many countries may have increased awareness and/or social approval bias. Lastly, data on weight and height were self-reported.

## 5. Conclusions

This study’s results suggest that the eating habits of adolescents may be influenced by the family context in which they live, and especially by its structure.

It is therefore useful to undertake targeted actions, such as the introduction of meetings on food education in schools or the provision of information on good eating habits through leaflets in places where adolescents meet, such as gyms or in the offices of pediatricians and general practitioners. All these actions could raise awareness among adolescents and their parents/caregivers on the importance of adopting healthy lifestyles as soon as possible [40,41].

## Figures and Tables

**Table 1:** Characteristics of the study population (*n* = 3210).

Variable	%	*n*
**Female**		
	49.7	1594
**Age categories**		
11 years	27.3	877
13 years	27.4	879
15 years	23.4	751
17 years	21.9	703
**BMI *z*-score**		
Mean	20.1	
SD	3.6	
Underweight	13.4	412
Normal weight	72.8	2238
Overweight	13.8	424
**Family structure**		
Both parents	80.5	2564
Single mother	10.9	348
Single father	1.4	45
Mother and stepfather	2.6	83
Father and stepmother	1.2	37
Other family structure *	3.4	107
**FAS**		
Low	19.6	612
Middle	59.3	1850
High	21.1	657
**Siblings**		
No siblings	25.5	817
Have at least one sibling	74.5	2391

FAS, Family Affluence Scale status. * Other family structures include grandparents and adults other than parents, such as foster parents, or care homes.

**Table 2:** Frequency of food intake by age groups (*n* = 3207).

	11 Years		13 Years		15 Years		17 Years	
	%	*n*	%	*n*	%	*n*	%	*n*
**Fruit consumption**								
Never	3.4	30	4.8	42	4.5	34	5.8	41
Less than once a week	4.7	41	6.6	58	8.8	66	8.3	58
Once a week	6.9	99	10.0	88	13.2	99	12.2	86
2–4 days a week	11.3	237	29.2	256	29.2	219	26.9	189
5–6 days a week	27.1	134	13.1	115	14.1	106	11.1	78
Once a day	15.3	158	17.2	151	15.4	116	20.5	144
Every day, More than once	18.0	177	19.0	167	14.8	111	15.2	107
**Vegetable consumption**								
Never	6.9	60	6.5	57	5.6	42	4.0	28
Less than once a week	7.7	67	5.4	47	6.3	47	4.1	29
Once a week	12.5	109	7.9	69	8.4	63	9.2	65
2–4 days a week	24.8	217	27.2	238	31.3	235	31.0	218
5–6 days a week	17.6	154	19.4	170	18.4	138	17.9	126
Once a day	15.0	131	18.4	161	15.7	118	15.1	106
Every day, More than once	15.7	137	15.2	133	14.3	107	18.6	131
**Sweets consumption**								
Never	5.3	46	3.3	29	4.0	30	4.1	29
Less than once a week	11.3	99	8.5	74	11.5	86	12.5	88
Once a week	15.5	136	15.4	135	14.9	112	14.2	100
2–4 days a week	30.7	269	30.1	263	32.8	246	29.6	208
5–6 days a week	13.5	118	17.6	154	14.6	110	15.2	107
Once a day	14.4	126	14.9	130	12.5	94	13.8	97
Every day, More than once	9.4	82	10.3	90	9.7	73	10.5	74
**Sugar-added soft drink cosumption**								
Never	17.8	156	16.5	144	17.3	130	22.5	158
Less than once a week	22.1	194	22.5	197	22.9	172	23.3	164
Once a week	27.2	238	25.0	219	23.2	174	21.9	154
2–4 days a week	17.6	154	21.3	186	20.2	152	18.8	132
5–6 days a week	8.0	70	5.8	51	8.3	62	5.1	36
Once a day	3.4	30	3.1	27	4.1	31	3.1	22
Every day, More than once	3.9	34	5.8	51	4.0	30	5.3	37

**Table 3:** Adjusted and unadjusted models for associations between family structure and adolescents’ food habits.

	Fruit Consumption (*n* = 3182)		Vegetable Consumption (*n* = 3178)		Sweets Consumption (*n* = 3180)		Soft Drink Consumption (*n* = 3180)	
	OR	95% CI	OR	95% CI	OR	95% CI	OR	95% CI
**Crude model family structure**								
Single mother	**0.63**	0.52–0.77	**0.64**	0.52–0.78	1.08	0.88–1.32	**1.22**	1.00–1.50
Single father	1.00	0.59–1.70	0.72	0.43–1.22	0.72	0.42–1.22	1.07	0.64–1.77
Mother and stepfather	0.75	0.51–1.11	0.83	0.57–1.22	0.79	0.54–1.16	**1.56**	1.06–2.29
Father and stepmother	**0.52**	0.29–0.91	0.68	0.37–1.22	0.88	0.50–1.54	1.70	0.94–3.07
Other family structure	1.02	0.73–1.43	**0.56**	0.40–0.79	1.04	0.75–1.46	1.35	0.95–1.93
**Adjusted model**								
Female	0.97	0.85–1.10	**0.64**	0.56–0.73	**0.87**	0.76–0.99	**1.55**	1.36–1.76
Age categories								
13 years	0.88	0.74–1.04	**1.24**	1.04–1.48	**1.33**	1.12–1.59	1.10	0.93–1.32
15 years	**0.67**	0.56–0.81	1.07	0.88–1.29	**1.23**	1.02–1.49	1.14	0.94–1.38
17 years	**0.77**	0.63–0.93	**1.28**	1.05–1.56	**1.31**	1.07–1.60	0.92	0.76–1.13
BMI	0.99	0.92–1.06	**1.13**	1.05–1.21	**0.77**	0.72–0.83	**0.91**	0.85–0.97
Family structure *								
Single mother	**0.68**	0.55–0.83	**0.66**	0.53–0.81	1.07	0.87–1.32	**1.33**	1.08–1.64
Single father	1.05	0.61–1.80	0.76	0.45–1.29	0.72	0.42–1.24	1.07	0.64–1.78
Mother and stepfather	0.75	0.50–1.11	0.80	0.53–1.19	0.81	0.55–1.20	**1.53**	1.02–2.28
Father and stepmother	**0.48**	0.26–0.87	o.75	0.40–1.41	0.95	0.52–1.75	1.73	0.89–3.36
Other family * structure	0.96	0.68–1.37	**0.68**	0.47–0.98	1.13	0.79–1.62	1.18	0.81–1.72
FAS **								
Middle	**1.27**	1.07–1.51	**1.39**	1.17–1.65	1.07	0.90–1.27	0.89	0.76–1.13
High	**1.56**	1.27–1.92	**1.59**	1.30–1.95	**0.78**	0.63–0.96	0.93	0.75–1.14
At least one sibling	1.03	0.89–1.20	0.89	0.77–1.04	0.93	0.81–1.08	1.06	0.92–1.23

FAS, Family Affluence Scale status. The following reference categories were used: gender, male; age, 11 years old; family structure, living with both parents; FAS, low SES. * Other family structure includes grandparents and adults other than parents, such as foster parents, or care homes. ** refers to summary score on Family Affluence Scale. In bold are the significant results.

## Data Availability

Data presented in this study are available in accordance with the 2022 Italian HBSC data access policy. Requests should be directed to the Italy Principal Investigator, Prof. Giacomo Lazzeri: giacomo.lazzeri@unisi.it.
